# Refugee Reception Re-examined: a Quantitative Study on the Impact of the Reception Period for Mental Health and Host Country Language Proficiency Among Syrian Refugees in the Netherlands

**DOI:** 10.1007/s12134-021-00820-6

**Published:** 2021-03-28

**Authors:** Roxy Elisabeth Christina Damen, Jaco Dagevos, Willem Huijnk

**Affiliations:** 1 Erasmus School of Social and Behavioural Sciences, Erasmus University Rotterdam, Burgermeester Oudlaan 50, 3062 PA Rotterdam, the Netherlands; 2 Erasmus School of Social and Behavioural Sciences, Erasmus University Rotterdam, Burgermeester Oudlaan, 50, 3062 PA Rotterdam, the Netherlands; 3 The Netherlands Institute for Social Research, Bezuidenhoutseweg, 30, 2594 AV, Den Haag, the Netherlands; 4 The Netherlands Institute for Social Research, Bezuidenhoutseweg 30, 2594 AV, Den Haag, the Netherlands

**Keywords:** Refugee integration, Early integration, Reception period, Mental health, Host country language proficiency, Syrian refugees

## Abstract

In many European countries, refugees spend their first period after arrival in the receiving country in reception centers. Though this reception period has been heavily criticized, especially in relation to mental health, few scholars examined its impact on refugee integration. Since host country language learning is the main focus for most recent arrivals, this study re-examines the impact of the (renewed) reception period on both refugee mental health and host country language proficiency. Using a unique dataset including 3209 Syrian permitholders in the Netherlands, we test a structural equation model to examine those relations directly and indirectly. Results partly replicate previous findings, showing the negative impact of the number of relocations on mental health as well as the negative impact of both length of stay and relocations on host country language proficiency. Nevertheless, we did not find support for a negative relationship between length of stay and mental health. Moreover, the image of the reception period is not as gloomy as before; activities asylum seekers can engage in during their stay is positively related to both mental health and host country language proficiency. The relationships between both relocations and activities and host country language proficiency can partially be explained by mental health. These results indicate that the reception period can be seen as both an obstacle and a facilitator. Since the context of reception is a post-migration stressor, these findings should inform receiving societies and inspire them to accommodate their refugee reception accordingly, ensuring a smooth(er) start for future refugees.

**Supplementary Information:**

The online version contains supplementary material available at 10.1007/s12134-021-00820-6.

## Introduction

Investigating potential obstacles and facilitators concerning integration among refugee groups has been of continuous academic, political, and societal interest. Though there is a large body of research on migrants’ integration, findings may not be easily transferred to refugees, who face particular challenges. In addition to loss and lack of resources all migrants experience, refugees might have been exposed to traumatic events before and after resettlement (Porter & Haslam, 2005; Bogic et al., 2015; Zimmerman et al., 2011). Consequently, previous research has shown the existence of the “refugee entry effect”: due to forced migration and the context of reception, refugees start their life in the host country at a disadvantage compared with other migrants (Bakker et al., 2017; Connor, 2010).

Though often especially disadvantageous for refugees due to their increased vulnerability, several studies have shown the importance of the first years after arrival in enabling further integration. Ghorashi (2005) claims that the first years in the receiving country are potentially the most essential for future success. Stevens (1999) illustrates this in her study by showing the learning curve tends to be steep during the first years after arrival and foundations for further improvements are laid.

In many European societies, refugees’ first period after arrival in the receiving country is characterized by stays in reception centers, in which asylum seekers must stay pending a decision on their asylum request. During this reception period, refugees have to deal with lost resources (Hobfoll, 2001) and uncertainties. Previous studies have shown that the institutionalized environment of the reception center allows residents to become passive, bored, and hopeless (Barclay et al., 2003; Ghorashi, 2005; Larruina & Ghorashi, 2016; Smets et al., 2017). Not surprisingly, the reception period has been heavily criticized for previous refugee groups in different countries, especially in relation to mental health (for a literature review, see Ryan et al., 2009) and to some extent labor market integration (Bakker et al., 2014; Hainmueller et al., 2016; Hvidtfeldt et al., 2018).

Despite the critiques, there exists little evidence regarding the impact of the reception period on early refugee integration. We do know that the length of the reception period often has an adverse effect on refugee mental health (Brekke, 2010; Steel et al., 2011; Keller et al., 2003), which can subsequently impede integration (Phillimore, 2011; Walther et al., 2019). Moreover, we know that language learning is central for most permitholders during the first years after arrival (Miltenburg & Dagevos, 2019; Ryan et al., 2008), and, like safety and stability (Shaw et al., 2020) can be regarded as key resettlement outcome, especially during the early stages after arrival. At the same time, like mental health, language proficiency can be regarded as a facilitator of further integration (Ager & Strang, 2008). In this regard, host country language proficiency has been described as a prerequisite for economic, political, social, and cultural integration (Hou & Beiser, 2006; Van Tubergen & Kalmijn, 2009)^1^. Because of the importance of the first period after arrival in the receiving country, we aim to re-examine the impact of the reception period for both mental health and host country language proficiency in this study. This is important, as both are characteristic for the first period in the receiving country, as well as crucial in enabling further integration.

We believe re-examining the impact of the reception period is relevant for various reasons. First, while the literature on the reception period is bleak, the criticism in the Netherlands often relates to the situation of refugees who arrived during the late nineties. At that time, prior to the 2001 Aliens Act^2^, it could take years before a final decision was reached on an asylum request, which resulted in a lengthy reception period (Bakker et al., 2014), while there were little to no opportunities for activities. Yet, there have been some changes regarding the reception period; the length of stay is often a lot shorter nowadays and there are more opportunities for activities^3^ (Bakker et al., 2018; Weeda et al., 2018). By re-examining the impact of the reception period, it can become clear whether reception centers still have predominantly negative consequences for its residents. Second, we employ a large dataset on Syrian permitholders in the Netherlands, which has been collected shortly after respondents’ stay in the reception center(s). This is beneficial since respondents can state their experiences more precisely and all respondents still live in the Netherlands^4^, which enables us to assess the impact of the reception period more accurately than before.

In order to re-examine the impact of the reception period, we include various aspects simultaneously. While most previous studies focused exclusively on the length of stay (Brekke, 2010; Hallas et al., 2007; Laban et al., 2004), we additionally focus on the number of relocations (see also Goosen et al., 2013; Nielsen et al., 2008 for studies on refugee children) and activity respondents engaged in during their stay in the reception center(s) (see also, Bakker et al., 2018; Weeda et al., 2018).

We study Syrian permitholders in the Netherlands, as they form by far the largest group of refugees that have arrived during the past years, after an estimated 13 million Syrians had to flee their homes since the outbreak of the civil war in the spring of 2011. Most displaced Syrians live in neighboring countries but about 1 million displaced Syrians have fled to Europe (Pew Research Center, 2018). In the Netherlands, more than half of the refugees whom received a (temporary) residence permit were of Syrian origin in 2014, rising to more than 70% of all permitholders in 2016. Nowadays, almost 100,000 Syrians reside in the Netherlands (CBS, 2019).

The question that arises in this study is: “What is the impact of the reception period on the mental health and host country language proficiency of Syrian permitholders in the Netherlands?” Answering this question is crucial to understand the role of Dutch refugee reception in facilitating early integration. In doing so, the current study contributes to the existing body of research in various ways. First, we provide insight in refugees’ first phase after arrival by focusing on the impact of the (renewed) reception period on both mental health and language proficiency, directly and indirectly. Second, due to the nature of our data, we can estimate these relationships more accurately than before. Third, we simultaneously examine three aspects of the reception period among a relatively new group of refugees^5^. Finally, while the impact of the reception period is scientifically relevant, it is also interesting for policy; while pre-migration stressors are largely fixed, post-migration stressors such as the reception period might be addressed through interventions upon resettlement.

## Theoretical Framework

### The Reception Period and the Dutch Context

Though the context of reception is unique for refugees, there is some variation regarding reception facilities across Europe (European Commission, 2001; AIDA, 2019). While most northern European countries tend to employ highly centralized state-sponsored programs, most countries in the south tend to provide minimal assistance. Yet, a large share of European states put emphasis on centralized reception centers^6^.

In the Netherlands, asylum seekers^7^ have to register at the central registration center and are then provided accommodation in an asylum-seeker center^8^ until their application is granted and social housing is available. Asylum seekers are randomly allocated to one of the centers, which are usually situated in rural areas^9^. If refugees want to leave the center or have visitors, this is subject to reporting to reception center staff (COA, 2016). Moreover, there are restrictions when it comes to working (voluntary or paid). Yet, in 2016, the Ministry of Social Affairs and Employment and the Central Organization for the Reception of Asylum Seekers (COA) committed to providing opportunities for various activities in the centers (Bakker et al., 2018). Once asylum seekers receive their permit to stay, the state provides them with social housing, usually in the same region as the center.

Though widely used across Europe, there has been increasing critique on the use of reception centers and the reception period in general. The main point of critique is that there is a risk that “the welfare system transforms active adult refugees into passive clients” (Ghorashi, 2005, p. 193; Harrell-Bond, 1999, p. 151; Wahlbeck, 1997, p. 101). While the integration process is understood as starting upon arrival, the reception period is sort of a stand-by situation, in which refugees cannot settle down and participate in the receiving society yet. Diken (2004) refers to reception centers as “non-places,” in which asylum seekers lead a life in a permanent state of exclusion. In line with this, the reception period has also been referred to as a form of social exclusion, which occurs when asylum seekers are physically separated from the host society (Robinson & Andersson, 2003). Reception centers in the Netherlands have been identified as a “total institution” in the past (ACVZ, 2013; Ghorashi, 2005; Larruina & Ghorashi, 2016; Smets et al., 2017): a place where individuals live apart from society and where their lives are regulated (Goffman, 1961).

### (Early) Integration; Mental Health and Language Proficiency

There is a rich body of literature concerning the concept of integration (see Castles et al., 2002, for an overview). Nowadays, most scholars agree that integration is a multidimensional, two-directional process, starting upon arrival in the receiving society (Berry et al., 2006; Schwartz et al., 2010). This means that multiple dimensions can be distinguished when it comes to integration and that integration is process that requires from immigrants a willingness to adapt to the host country (Ager & Strang, 2008; Lomba, 2010; Phillimore, 2011) and from the host country a willingness to facilitate integration and accept newcomers (Castles et al., 2002).

Previous studies have labeled health as one of the functional dimensions of integration (Ager & Strang, 2008). In other words, poor mental health makes it more difficult to actively participate in society (Phillimore, 2011; Walther et al., 2019). Several studies have shown that incidence rates of mental and physical health problems are relatively high among refugees (Beiser, 2006; Casado & Leung, 2002; Gerritsen et al., 2006). The share of Syrian permitholders in the Netherlands struggling with mental health issues is also rather large compared to the general population (Uiters & Wijga, 2018). While on the one hand possibly being formed or reinforced during the reception period, mental health problems can in turn form a barrier to integration.

For most permitholders the first period in the receiving society is largely devoted to learning the host country language, as this is a vital resource relevant to the host society (Hou & Beiser, 2006; Ryan et al., 2008; Van Tubergen & Kalmijn, 2009) and a facilitator of integration (Ager & Strang, 2008). Numerous studies have shown the importance of language proficiency in enabling social integration (Fong & Isajiw, 2000; Martinovic et al., 2009), labor market integration (Chiswick & Miller, 2001; Shields & Price, 2002), and belonging (Amit & Bar-Lev, 2015; Walters et al., 2007). Qualitative studies also showed that language is perceived to be key by refugees themselves when it comes to integration (Earnest et al., 2015; Wallin & Ahlström, 2005; Damen et al., 2019; van Liempt & Staring, 2020).

During the first period after arrival in the receiving country, language is regarded as one of the most importance resources to acquire. Later, language skills are crucial in fostering participation in other domains (Portes & Rumbaut, 2006) and will be more of a means. While characteristic for the first period after arrival, in time, both mental health and language proficiency can thus be considered a prerequisite for acquiring other types of resources. Since we understand integration as a multidimensional two-directional process, we will not only analyze mental health and host country language proficiency of Syrian permitholders to say something about their integration, but we will focus specifically on the impact of the reception period which can be seen as a postmigration stressor (see also, Berry et al., 1987) rooted in the receiving society.

### The Reception Period and Mental Health

The focus of mental health research among refugees has been on the impact of pre-migration trauma. But, while pre-migration trauma does predict mental disorders and PTSD, the post-migration context can also play a role in determining mental health (Li et al., 2016; Porter & Haslam, 2005). As a post-migration stressor, the reception period has regularly been associated with poor mental health. Statements regarding this relationship are often in line with the highly regulated living environment (Barclay et al., 2003). This environment allows residents to become passive, bored, and hopeless; (Barclay et al., 2003; Ghorashi, 2005; Larruina & Ghorashi, 2016; Smets et al., 2017); residents are thereby institutionalized, and the self-esteem and self-determination diminishes (ACVZ, 2013; Larruina & Ghorashi, 2016). Also, the lack of privacy in an asylum center can exacerbate mental health problems (Laban et al., 2004; Ghorashi, 2005).

Most studies investigating the reception period and mental health have focused on the length of stay, showing that the duration of the asylum procedure is an important risk factor (Hallas et al., 2007; Laban et al., 2004), among both young (Brekke, 2010) and adult refugees (Steel et al., 2011; Keller et al., 2003). The isolated life in the reception center causes refugees to waste potentially the most vital years of their lives (Ghorashi, 2005). The longer people live in such a way, the more likely that this will have an impact on one’s mental health. We expect a longer stay in the reception center to have negative consequences for the mental health of Syrian permitholders in the Netherlands (**H1**).

Apart from the impact of length of stay, few studies investigated the role of relocations. Outside of the reception context, Mulvey and Council (2013) found that refugees of age sixteen and over experienced moving neighborhood as very disruptive to their sense of belonging. Relocations between reception centers are mostly unpredictable and arranged through institutions, which can contribute to mental health problems because of its disruptiveness (Vitus, 2011; Mulvey & Council, 2013). Generally, scholars that did include this aspect of the reception period focused on asylum-seeking children, showing that a high relocation rate (Goosen et al., 2013) and four or more relocations (Nielsen et al., 2008) are associated with a higher risk of mental problems. We expect a similar relationship for adults: a higher number of relocations between centers will have negative consequences for the mental health of Syrian permitholders in the Netherlands **(H2)**.

As mentioned earlier, the bleak image on the reception period may no longer be justified. As of recently, there are various opportunities for engaging in activities—such as language learning and volunteering—while living in a reception center in the Netherlands^10^ (COA, 2018). Engaging in such activities can result in the feeling of regaining control over one’s life, help to (re)gather resources, provide distraction, a goal, and increase confidence (Bakker et al., 2018; Hobfoll, 2001; Ryan et al., 2008). This relatively new aspect of the reception period could make an important difference by breaking the circle of passivity, which may be positively related to mental health (ACVZ, 2013). Based on these claims we expect that, despite the negative stigma regarding the reception period, participating in activities during the reception period has positive consequences for the mental health of Syrian permitholders in the Netherlands (**H3**).

### The Reception Period and Language Proficiency

Though we understand integration as a multidimensional two-directional process, there is relatively little consideration of the impact of receiving societies on the lives of forced migrants within refugee integration studies (Morville et al., 2015). As conditions of the asylum procedure and reception period are rooted in receiving society, the reception period is pre-eminently suited to study as a receiving society condition in relation to early integration. Though there are some studies that have focused on the impact of the reception period on labor market integration (Bakker et al., 2014; Hainmueller et al., 2016; Hvidtfeldt et al., 2018), few studies related the reception period to host country language learning.

The time refugees spend in a reception center can have important consequences for their language learning. Language acquisition requires initiative, motivation and perseverance, while reception policy did not use to encourage these characteristics (ACVZ, 2013). The longer people live in such a way, the harder it becomes to acquire post-migration human capital (de Vroome & van Tubergen, 2010). Besides, while staying in a reception center, the opportunities for asylum seekers to establish contacts with natives are constrained, leading to less exposure to the host country language (Van Tubergen, 2010). Consequently, a longer stay in a reception center is negatively associated with host country language proficiency (idem). Moreover, de Vroome and van Tubergen (2010) showed that especially human capital (including host country language proficiency) is negatively affected by a lengthy stay. We expect a longer stay in the reception center to have negative consequences for the host country language proficiency of Syrian permitholders in the Netherlands (**H4**).

Though no empirical evidence exists for a direct relationship between the number of relocations and host country language proficiency, we argue a similar negative relationship can be expected for more relocations as for a lengthy stay. Relocations are disruptive (Goosen et al., 2013; Nielsen et al., 2008; Vitus, 2011), and if one has to relocate often, it could be difficult to engage in meaningful social relations with natives, leading to less exposure to the native language. Moreover, if one has to relocate often this could be an obstacle to engaging in activities and (re)gain resources. Based on these arguments, we expect more frequent relocations to have negative consequences for the host country language proficiency of Syrian permitholders in the Netherlands (**H5**).

In order to integrate into the receiving society, refugees need to (re)gain lost resources and acquire new skills relevant to the host society (Hobfoll, 2001; Ryan et al., 2008). (Re)gaining such resources can already start during the reception period, by means of participating in society through engaging in activities. Engaging in such activities can improve host country language proficiency in various ways. First, through activities such as (volunteer)work, asylum seekers can experience more exposure to Dutch natives, with whom they can practice and improve their language skills (see also, Bakker et al., 2018). Second, in some reception centers there is the possibility to take language courses or learn the language by yourself. By starting to learn the host country language already in the center, language proficiency can improve quicker. So, engaging in activities during the reception period is also expected to be directly related to language proficiency, but in contrast to length of stay and relocations, we expect engaging in activities during the reception period to have a positive impact on host country language proficiency of Syrian permitholders in the Netherlands (**H6**).

### Mental Health and Language Proficiency

Next to the direct relations between the three aspects of the reception period and both mental health and language proficiency, we argue that mental health can also be related to language proficiency. There has been little research into the relationship between mental health and host country language proficiency among refugees. However, Steel et al. (2011) mention that several studies did demonstrate a link between the severity of PTSD and depressive symptoms and cognitive and memory functioning which, in turn, may interfere with host country language acquisition (see, among others, Beiser & Hou, 2001; Hinton et al., 1997; Söndergaard & Theorell, 2004; Ward, 2002). Health is considered a prerequisite for acquiring other types of resources, and host country language is an important resource to gain in the first years after arrival. Thus, being mentally stable can be expected to be related to better host country language proficiency (**H7**).

**Fig. 1:**
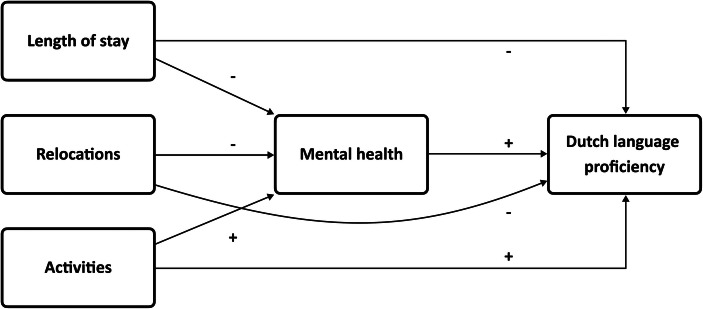
Conceptual path model showing the hypothesized paths and the expected direction of the relationships

The asylum process can be anti-integrative in the sense that the combination of uncertainty, passivity, social isolation, and poor access to services can have long-term impacts upon mental health and then affect access to wider integration (Phillimore, 2011; Walther et al., 2019). For example, it is possible that social isolation or diminished participation among refugees in reception centers may impact language acquisition also through mental health (Clarke et al., 1993). Thus, if there is an association between conditions of the reception period and mental health, it can be expected that mental health is a mediating variable between the reception period and language proficiency among Syrian permitholders in the Netherlands (**H8**). Figure 1 shows the conceptual model including the hypothesized paths and the expected direction of the relationships.

## Data and Methods

This analysis was based on data of the survey “New Permitholders in the Netherlands” (NSN2017), conducted at the request of four Dutch ministries and part of a larger project (Longitudinal cohort study permitholders) aiming to gain insight into early integration of refugees. The project is unique as the survey includes extensive information on pre-migration, migration, and post-migration characteristics as well as information on refugee integration collected shortly after arrival.

The survey was carried out in 2017^11^ among Syrians aged 15 and older who received a (temporary) residence permit in the Netherlands between January 1, 2014, and July 1, 2016 (coinciding with the period of high influx of asylum seekers in the Netherlands, which peaked in 2015). Their children born in the Netherlands and family members who reunited in 2014–2015 also belong to the target population. A single random cluster sample was drawn from the target population by Statistics Netherlands. Next, the questionnaire was tested thoroughly and translated into Modern Standard Arabic. A sequential mixed mode survey design was used; respondents were first invited to complete the survey online (CAWI), but if they did not, they were given the opportunity to complete the survey in person with an interviewer (CAPI). In case of no response, interviewers would visit respondents up to four times to make an appointment for participation^12^. All interviewers spoke Arabic and were from the same ethnic background as the respondents.

In total, 3209 Syrians completed the survey, corresponding to a response rate of 80.6%. The high response rate can partly be attributed to the personal and repetitive approach, but it also shows people were eager to tell their story. Lastly, the survey file was weighted by Statistics Netherlands to match the distribution in the sample with that in the population. For this study, respondents who indicated never to have lived in a reception center (*N = 186*) were excluded. Consequently, the final sample consisted of 3023 respondents. The sampling, extensive fieldwork, bilingual interviewers, high response rate, and weighting of the data resulted in a unique and high-quality data set.

### Measures

*Host country language proficiency*—in this case proficiency in Dutch—was included as a latent variable measured by three items: “If you have a conversation in Dutch, do you often, sometimes or never have trouble with the Dutch language?”, “Do you often, sometimes or never have trouble understanding the Dutch language when reading newspapers, letters or brochures?”, and “Do you have trouble writing in Dutch?” Respondents could choose from three categories: (1) yes, often; (2) yes, sometimes; and (3) no, never. Each of the items loaded highly on the designated factor, with loadings ranging from .703 to .868. A higher score indicated being more proficient in the host country language (α = .735)^13^.

*Length of stay in the reception center(s)* was captured by the number of months one stayed in such center(s). Respondents were asked: “How long have you lived in an asylum seekers’ center/asylum seekers’ centers all together? Enter the number of months.” This variable was included as continuous variable, a higher score indicated a longer stay in reception center(s).

The *number of relocations* was measured by the question: “How many different asylum seekers’ centers have you lived in?” Since we were interested in the number of relocations, we subtracted 1 from the number of different reception centers. For example, a respondent who has lived in two different centers has had one relocation. This variable was included as a continuous variable, a higher score indicated more relocations.

To measure *activities during the reception period* respondents were asked “Did you do one or more of the following activities during your stay in the asylum seekers center? (1) followed Dutch language lessons, (2) learned Dutch yourself, (3) volunteered, (4) did paid work and (5) followed training or education.” This variable was included as a sum score, adding up all activities one engaged in.

To assess respondents’ *mental health*, the Mental Health Inventory 5 (MHI-5) was used. The MHI-5 is a measuring instrument that gives an impression of people’s mental health in the last 4 weeks (Rumpf et al., 2001), at the time of the survey. Respondents were asked how often they felt: very nervous, depressed and gloomy, calm, so bad that nothing could cheer you up and happy. They could answer these questions on a 6-point scale, ranging from (1) constantly to (6) never. Answers to the items calm and happy were reversed. For each person, the values of all five questions were recoded to a 0–5 scale and multiplied by 4, and next a sum score was calculated based on the five items. In this way the minimum score is 0 and the maximum score is 100. The higher the score, the better ones’ mental health.

Some individual background characteristics were included as control variables. As pre-migration factor previous education was included by considering *having attended higher education in Syria or another country abroad* as dichotomous variable. As post-migration factor, *length of stay in the Netherlands* was included as continuous variable indicating the length of stay in months. Moreover, some demographic characteristics were included. *Gender* was included as a dichotomous variable (0 = male, 1 = female). *Age* was included as continuous variable. Lastly, *having a partner in the household, having children in the household* and the *family being incomplete* were included as dichotomous variables.

### Method of Analysis

To test the hypotheses, structural equation modelling (SEM) was used (Mplus version 8). The SEM framework integrates simultaneous equation models and confirmatory factor analytical models (CFA) (Kline, 2010), which allows us to test mediation hypotheses with latent variables. Because the “host country language” items were not normally distributed, models were fitted using weighted least squares means and variance adjusted estimation (WLSMV) as a method of parameter estimation. This estimator is recommended for the analysis of categorical dependent variables (Flora & Curran, 2004; Muthén et al., 1997). Missing values^14^ were imputed by using multiple imputation in Stata (version 15).

## Results

Table 1 shows the correlations between the core constructs^15^. All the associations are in the expected direction and almost all are significant. The correlation between the length of stay in the reception center and mental health is not significant. It can furthermore be observed that there are strong positive correlations between the three aspects of the reception period. The VIF score was below 1.3 for all the constructs, which is why we can be sure that there is no multicollinearity problem in this analysis.

**Table 1:** Correlations between the main constructs, (*N* = 3023)

	1	2	3	4	5
1. Host country language proficiency^a^	-				
2. Length of stay reception	−0.113***	-			
3. Number of relocations	−0.096***	0.424***	-		
4. Number of activities	0.274***	0.211***	0.142***	-	
5. Mental health	0.112***	−0.007	−0.040*	0.044*	-

^a^Host country language proficiency is a latent construct. * *p* < 0.05; ** *p* < 0.01; *** *p* < 0.001 (two-tailed)

### Explaining the Impact of the Reception Period on Mental Health and Language Proficiency

**Fig. 2:**
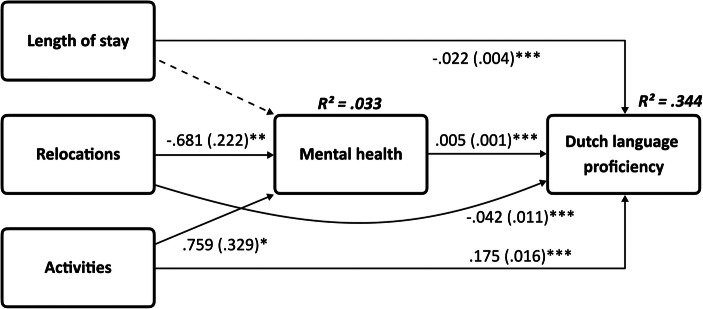
A path model explaining the relationship between characteristics of the reception period, mental health and host country language proficiency among Syrian permitholders in the Netherlands (*N* = 3023). Unstandardized coefficients with standard errors in the parenthesis. Nonsignificant paths are presented as dashed lines. *p* < .05; ** *p* < .01; *** *p* < .001.)

A structural equation model was estimated with the three aspects of the reception period as independent predictors of host country language proficiency and mental health as mediator of this relationship. Gender, age, and household characteristics, having attended higher education abroad and length of stay in the Netherlands, were included as control variables. The full model including control variables fitted the data well (χ^2^(22) = 96.767, *p* = .000, CFI = .979, TLI = .955, RMSEA = .034). Ideally the Chi-square value should be insignificant; however due to the large sample size, it is sufficient to look at the other fit statistics (Kline, 2010, p. 198). We did not conduct post hoc modifications because of the good fit of the data to the model (MacCallum et al., 1992). The findings related to the hypothesized paths are presented in Fig. 2. Paths from the control variables gender, age, household characteristics, higher education, and length of stay in the Netherlands were accounted for in the model but not reported in the figure. Estimates for the control variables can be found in Online Resource 1.

There was no significant relationship between the length of stay in the reception center and refugee mental health, thereby rejecting H1. The number of relocations was negatively related to refugee mental health, and activities were positively related to refugee mental health, confirming H2 and H3. Furthermore, a longer stay in the reception center and more frequent relocations were negatively related to host country language proficiency, while activities in the reception center were positively related to host country language proficiency, confirming H4, H5, and H6. Additionally, refugee mental health was positively related to host country language proficiency, confirming H7.

Regarding the mediating role of mental health between the characteristics of the reception period and host country language proficiency, a significant indirect effect was found from relocations to host country language proficiency via mental health, *β* = −.004, SE = .001, β* = -.007, *p* = .006, and a significant indirect effect from activities to host country language proficiency via mental health, *β* = .004, SE = .002, β* = .005, *p* = .031. A significant direct path did remain from both relocations (*β* = −.042, SE = .011, β* = −.080, *p* < .001) and activities (*β* = .175, SE = .016, β* = .219, *p* < .001), indicating partial mediation. There was no significant indirect effect for the length of stay, *β* = .000, SE = .000, β* = −.001, *p* < .653, thereby only partly confirming H8.

### Alternative Analysis

We checked whether our findings held when (1) including the self-reported rating (0–10) for host country language proficiency instead of the latent variable, (2) using the frequently used sum score of 60 points as a cut-off point (Driessen, 2011) instead of the MHI-5 sum score, (3) using a dichotomous variable for being active (engaging in one or more activities) or passive (no activities at all), (4) including separate dummies for the activities during the reception period, (5) including a cut-off point for having stayed in reception nine months or longer, (6) including a cut-off point for being relocated three or more times, (7) including either length of stay or relocations, and (8) excluding family members who reunited based on a proxy. These alternative models yielded largely the same results (see Online Resources 2 to 9).

## Discussion and Conclusion

In many European countries, refugees spend their first period after arrival in the receiving country in reception centers. Previous research has been critical regarding the reception period, especially in relation to refugee mental health and labor market integration, showing a negative impact of a lengthy and monotonous reception period. However, little evidence exists regarding the impact of the reception period on early integration, and the critiques are often based on the situation of the past.

With this study conducted in the Netherlands among a representative sample of 3023 Syrian permitholders, we contributed to the existing literature in three ways: (1) we provided insight in Syrian refugees’ first phase after arrival by focusing on the impact of the (renewed) reception period on mental health and host country language proficiency, (2) due to the nature of our data we were able to estimate these relationships more accurately than before, and (3) we simultaneously examined three aspects of the reception period among a new group of refugees. We partly replicated results from earlier studies, showing the negative impact of some aspects of the reception period on both mental health and language proficiency. Yet, the image is not as gloomy as before, as the possibility to engage in activities during the reception period can have a positive impact for both mental health and language proficiency.

We did not find support for a relationship between the length of stay in the reception center(s) and Syrians’ mental health. A possible explanation for this is that Syrian refugees in general had a relatively short stay in reception centers as they spent an average of 9 months (Dagevos & Miltenburg, 2018), compared with over a year and a half for previous refugee groups (Bakker et al., 2014; Dourleijn & Dagevos, 2011). The length of stay can make a difference to the extent to which this period impacts mental health. Earlier studies demonstrate such a negative impact only to occur for people who have stayed in reception centers for over 5 years (Bakker et al., 2014) or more than 2 years (Laban et al., 2004). The negative impact of the length of the reception period may therefore not apply to the situation of the Syrians. However, we did find a negative relationship between the length of stay and host country language proficiency. While the stay might have been too short to impact mental health, the period in the reception center(s) remains a sort of stand-by mode. Residents remain relatively isolated which can delay their language acquisition (see also, Van Tubergen, 2010; de Vroome & van Tubergen, 2010).

We did find support for a negative relationship between the number of relocations and mental health. This result confirms those of previous studies showing a negative association between relocations and asylum-seeking children’s mental health (Goosen et al., 2013; Nielsen et al., 2008). For Syrian permitholders in the Netherlands, it might not so much be the length of the stay but the disruptive nature of the relocations (Vitus, 2011; Mulvey & Council, 2013) that impacts their mental health. The number of relocations was also negatively related to host country language proficiency. We expected this to be explained by difficulties to engage in social contacts or activities if one had to relocate often. However, a positive correlation was found between relocations and activities. Though asylum seekers are distributed across reception centers randomly, it could be that people who had to relocate often differ from those who had to relocate less on aspects we did not account for in this study.

Apart from the overall negative impact of the length of stay and relocations, we found support for a positive side to the reception period. Activities one can engage in during their stay in the reception center(s) was positively related to both mental health and host country language proficiency. Thus, being active in the first months of stay can be seen as an essential condition for thriving or coping (see also: Ghorashi, 2005). These activities can offer distraction, give purpose, and improve self-confidence (Bakker et al., 2018; Hobfoll, 2001; Ryan et al., 2008), which is beneficial for refugee mental health. In addition, these activities provide refugees with the opportunity to start (re)gaining lost resources and acquire new skills relevant to the host society (Hobfoll, 2001; Ryan et al., 2008). Engaging in such activities may improve host country language proficiency through exposure to natives (see also, Bakker et al., 2018; Van Tubergen, 2010) or by already starting to learn the host country language.

To explain the impact of the reception period on host country language proficiency, we included mental health as a mediator. We found a positive relationship between mental health and host country language proficiency, indicating mental health is a prerequisite for regaining other types of resources, such as the host country language. In addition, we found a significant indirect path for both relocations and activities, while a direct path remained. The relationships between relocations and host country language proficiency as well as activities and host country language proficiency can therefore be partially explained by mental health. This suggests that on the one hand the reception period can be anti-integrative in that the combination of uncertainty, passivity and poor access to services can have long-term impacts upon mental health and then affect wider integration (Phillimore, 2011; Walther et al., 2019). On the other hand, the way in which this period is filled in can have positive implications by counteracting passivity and offering distraction through activities so people remain mentally stable and can in turn make a smooth(er) start in the receiving country.

### Limitations and Directions for Future Research

The complete model explains about 34% of host country language proficiency, which shows that the three aspects of the reception period and refugee mental health are important for early integration, confirming the importance of the first period after arrival in the receiving country. Though, mental health is not explained well by the variables in the model. A possible explanation for this is that even though post-migration stressors can play a role in refugee mental health, pre-migration stressors could be far more important. Evidence from previous studies suggests that pre-migration experiences were the most consistent factors associated with poor mental health in both recently resettled and long-settled refugees (Chen et al., 2017; Bogic et al., 2015), while post-migration factors may moderate the ability to recover from pre-migration trauma (Chen et al., 2017; Hynie, 2018; Ghorashi, 2005). Besides, there are other post-migration stressors which may affect refugee mental health that we did not take into account, such as discrimination, not being able to find a job or social isolation. It would be interesting to include several forms of post-migration stressors in a future study on refugee mental health to estimate the impact of these different types of stressors. Moreover, mental health was measured using the psycho-social MHI-5 scale, yet it could be that we would better be able to explain refugee mental health if we would be able to additionally include biological factors as indicators of mental health (such as adaptation to new climate, new sun activity, new diet and new regime of physical activities). Though this was outside the scope of our study, we do encourage others to include such indicators in future research.

Additionally, the cross-sectional nature of our data can be seen as a limitation. Even though our expectations about the relations are based on theoretical grounds, some of the relationships can be reciprocal. For example, language barriers can significantly affect refugee mental health through the access to healthcare and (lack of) communication with healthcare providers (Hynie, 2018). Besides, not being able to speak the host country language can be a determinant of depression (Bogic et al., 2015). Thus, some caution is required when interpreting the associations as described. Yet, the items on the aspects of the reception period can be seen as rather exogenous, as in most cases they already took place before the survey was conducted. It could however be that if one was more mentally stable when entering the Netherlands, it would be more likely that one would engage in activities. In order to confirm this expectation, we would need information on the mental health of refugees before the reception period.

Another limitation of this study is that we have focused on Syrian permitholders in the Netherlands specifically. Because of differences in reception conditions, it might not be possible to generalize the results of this study to permitholders in other countries. Future studies could focus on other groups of refugees in different countries to see if similar relationships can be found. Moreover, it would be interesting to also learn if the relationships we have shown in this study differ within the group of Syrian newcomers, in order to say something about which groups can more easily handle the reception period than others and for whom additional assistance might be necessary.

Lastly, we used different measures for activities showing that engaging in various activities, being active versus passive (online resource 4) and especially language learning and going to school (online resource 5) are positively related to either mental health and/or host country language proficiency. Future studies could take a closer look at the content of these activities. By looking at the duration and intensity of the activities one engaged in during their time in the reception center(s), we could learn more about the value of such activities.

## Conclusion and Implications

In conclusion, the reception period has an impact on both mental health and language proficiency, as an obstacle and as a facilitator. Our study partly confirms the negative stigma surrounding the reception period but also puts it into perspective by showing a positive side. Moreover, mental health facilitates as a partial mediator between aspects of the reception period and host country language proficiency. On the one hand, the reception period can thus be seen as anti-integrative since lengthy stays and relocations can have long-term impacts upon language proficiency, either directly or also indirectly when it comes to relocations. On the other hand, the way in which this period is filled in can have positive implications, both directly and indirectly through mental health. These findings partly confirm the refugee entry effect (Bakker et al., 2017). Syrian permitholders in the Netherlands may indeed experience a delay in their integration due to the context of the reception period and their mental health. Nonetheless, the impact of the reception period might not be as negative when it is spent actively, and this refugee entry effect could be counteracted.

Since the context of reception is a post-migration stressor, it can be addressed by receiving societies through interventions upon resettlement. Taking the previous studies on this topic and our findings into account, receiving societies should review their policies regarding refugee reception and aim to prevent newcomers from becoming passive, hopeless, and isolated during the reception period, as this has major implications for both mental health, language proficiency, and further integration. As our results indicate, short stays, few relocations, and opportunities for activities during the reception period are beneficial for both permitholders and society. These finding should inform and inspire receiving societies to accommodate their refugee reception accordingly, contributing to a smooth(er) start of refugees having to settle in the Netherlands or elsewhere in the future.

### Supplementary Information

ESM 1 (PDF 482 kb)

## Appendix 1 - Descriptive statistics

**Table 2:** Descriptive statistics of the variables included in the main analysis (*N* = 3023)

	Range	Mean/proportion	S.E.
*Difficulties speaking Dutch*			
Often	0/1	0.388	-
Sometimes	0/1	0.529	-
Never	0/1	0.083	-
*Difficulties writing Dutch*			
Often	0/1	0.284	-
Sometimes	0/1	0.502	-
Never	0/1	0.214	-
*Difficulties reading Dutch*			
Often	0/1	0.380	-
Sometimes	0/1	0.535	-
Never	0/1	0.086	-
*Length of stay reception*	0–72	8.782	0.095
*Relocations*	0–8	2.626	0.034
*Activities*	0–5	1.768	0.022
*Mental health*	0–100	64.361	0.386
*Control variables*			
Age	15–75	33.183	0.218
Female	0/1	0.295	-
Child at home	0/1	0.512	-
Lives with partner	0/1	0.561	-
Family incomplete	0/1	0.113	-
Higher education abroad	0/1	0.299	-
Length of stay NL	6–84	27.025	0.140

## Footnotes

1 This does not mean that refugees cannot also contribute to different spheres of society without their ability to articulate in the host country language. Moreover, their own language can serve as cultural enrichment for the host society (Gojkov-Rajić & Prtljaga, 2013).

2 In the year 2001, a new Aliens Act was adopted with the main aim of shortening the asylum procedure.

3 The data we employ in this study was collected before the emerge of COVID-19. Due to the pandemic, asylum procedures were often put on hold resulting in lengthier stays and less opportunity for activities. Though it is beyond the scope of this study to draw conclusions on the impact of COVID-19, our results may signal to the possible consequences of these delays and restrictions.

4 There is thus no selection effect, as in Bakker et al. (2014) their study, for which the data was collected years later.

5 Weeda et al. (2018) included these three aspects but focused on social integration of refugees in Rotterdam.

6 In Belgium, the Netherlands, Sweden, Denmark, Finland, Germany, Switzerland, Poland, Slovenia, Croatia, Serbia, and Romania asylum seekers are initially provided with accommodation in reception centers (though the capacity and conditions vary per country). France, Austria, Spain and the UK all have several first-line reception centers, but these are not available to all asylum seekers. In Spain and the UK most asylum seekers have to find accommodation in private housing. Greece, Ireland, Italy, Portugal, Hungary and Bulgaria have very limited or no centralized reception facilities (AIDA, 2019).

7 An asylum seeker is a person applying for protection under the UNHCR Refugee Convention from 1951, while a refugee is a person whose claim for protection is recognized (UNCHR, 2006). Asylum seekers who are recognized as refugees will receive their refugee status. They are also called permitholders.

8 Since 1990, the semi-governmental organization COA (Central Organization for the Reception of Asylum Seekers) is in charge of Dutch asylum seeker centers (Rast et al., 2019).

9 Due to the high influx of refugees in 2015, not all newcomers could be accommodated in regular centers, so some had to stay in emergency shelters (COA, 2015).

10 Regarding other European countries, those who provide accommodation in centers all provide some type of activities for residents, the type and frequency depending on the particular country or center. Mostly these are activities such as learning the host country language, while some provide different types of workshops or training (AIDA, 2019).

11 This was led by The Netherlands Institute for Social Research (SCP). There was collaboration with Statistics Netherlands (CBS), the Research and Documentation Center (WODC), the National Institute for Public Health and the Environment (RIVM), and research agency Labyrinth.

12 See (Kappelhof, 2018) for more details on the survey design and implementation.

13 This measurement provides a global indication of Dutch language proficiency as it is based on Syrians own assessment. Nevertheless, previous experiences with survey research among migrant groups show that this type of measurement provides plausible results in showing the differences in Dutch language proficiency within and between groups (Dagevos & Miltenburg, 2018; Dourleijn & Dagevos, 2011, p. 79).

14 There were missing values on “having a university degree from Syria or another country” (*N*=22) and “length of stay in the Netherlands” (*N* = 3).

15 Descriptive statistics can be found in Appendix 1.
