# Supplementary material for: Refugee Reception Re-examined: a Quantitative Study on the Impact of the Reception Period for Mental Health and Host Country Language Proficiency Among Syrian Refugees in the Netherlands
Source: J Int Migr Integr. 2021 Mar 28;23(1):1–21. doi: 10.1007/s12134-021-00820-6 (PMC8004562; doi:10.1007/s12134-021-00820-6)
Supplement: Supplementary file 1 — (PDF 482 kb) [file 12134_2021_820_MOESM1_ESM.pdf]

Online Resources

Title: Refugee reception re-examined: a quantitative study on the impact of the reception period for mental health and host country language proficiency among Syrian refugees in the Netherlands.

Journal: Journal of International Migration and Integration

Authors: Roxy Damen, Msc.* Prof. Dr. Jaco Dagevos and Dr. Willem Huijnk

* Corresponding author. Department of Sociology, Erasmus University Rotterdam, Burgermeester Oudlaan 50, 3062 PA Rotterdam, the Netherlands. E-mail: damen@essb.eur.nl

Online Resource 1 - *Estimates for the control variables included in the main analysis*

Table 1 Estimates for the control variables in the analysis (N = 3023), estimator WLSMV, standard errors in parenthesis.

	**Mental health**		**Host country language proficiency**	
Age	-0.047	(0.041)	-0.030	(0.002)***
Female	-4.994	(0.889)***	0.025	(0.044)
Child at home	-0.218	(1.194)	-0.019	(0.057)
Lives with partner	5.664	(1.220)***	-0.224	(0.060)***
Family incomplete	-4.679	(1.300)***	-0.093	(0.063)
Higher education abroad	-1.746	(0.873)*	0.403	(0.041)***
Length of stay NL	-0.038	(0.051)	0.020	(0.002)***

* p <.05; ** p <.01; *** p <.001.

Interpretation: Older Syrians reported weaker host country language proficiency, while women reported being less mentally stable compared to their male counterparts. Those who live with their partner in general reported being more mentally stable but weaker proficiency in the host country language as those not living with a partner. Those of whom their family is incomplete reported being less mentally stable. Syrians who engaged in higher education abroad reported being less mentally stable but stronger host country language proficiency as those who did not engage in higher education. Lastly, those who have stayed in the Netherlands longer reported stronger host country language proficiency.

Online Resource 2 – Alternative analysis including the self-reported rating for host country language

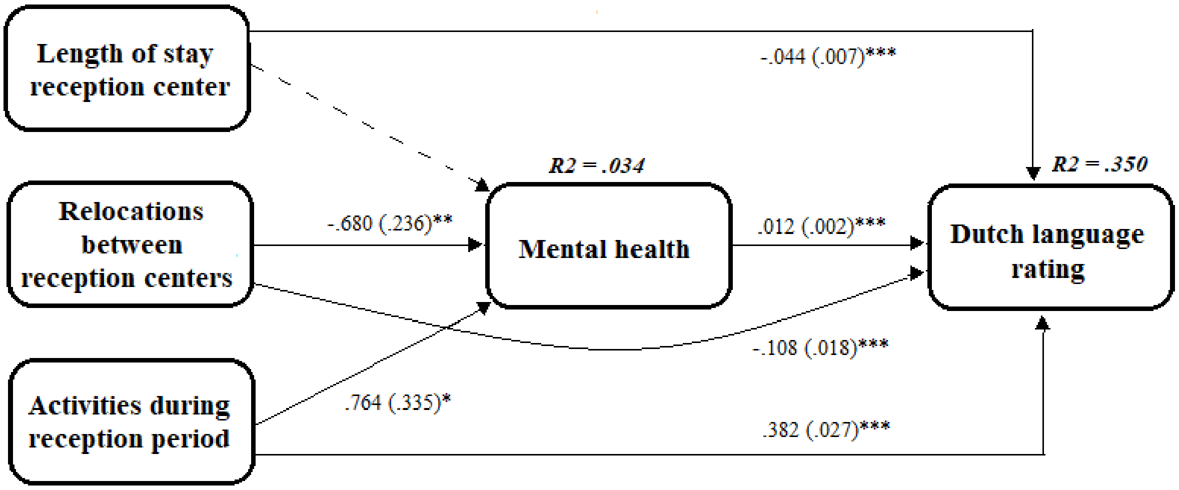

Figure A. A path model explaining the relationship between characteristics of the reception period and host country language rating for Syrian permitholders in the Netherlands (N = 3023). Unstandardized coefficients with standard errors in the parenthesis. Paths from the control variables gender, age, household characteristics, higher education and length of stay in the Netherlands were accounted for in the model but not reported in the figure and nonsignificant paths are presented as dashed lines. Estimator MLR, Model fit: (χ^2 (0) = 0.000, p = .000, CFI = 1.000, TLI = 1.000, RMSEA = .000). * p <.05; ** p <.01; *** p <.001

Online Resource 3 – Alternative analysis using a cut-off point for being psychologically unhealthy

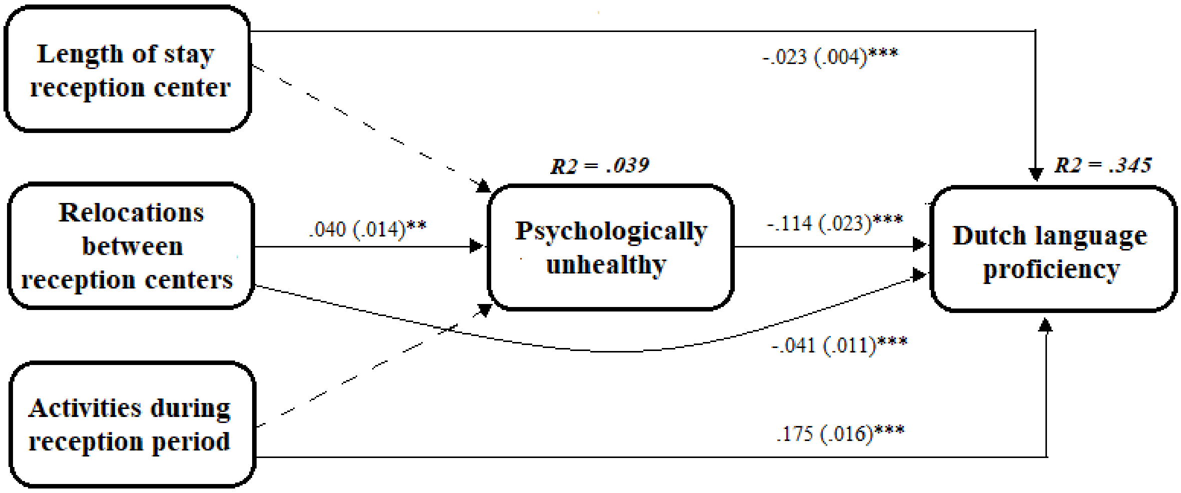

Figure B. A path model explaining the relationship between characteristics of the reception period and host country language proficiency for Syrian permitholders in the Netherlands (*N* = 3023). Unstandardized coefficients with standard errors in the parenthesis. Paths from the control variables gender, age, household characteristics, higher education and length of stay in the Netherlands were accounted for in the model but not reported in the figure and nonsignificant paths are presented as dashed lines. Model fit: (χ^2 (22) = 98.964, *p* = .000, CFI = .978, TLI = .954, RMSEA = .034). * *p* <.05; ** *p* <.01; *** *p* <.001

Online Resource 4 – Alternative analysis including a dichotomous variable for being active or passive

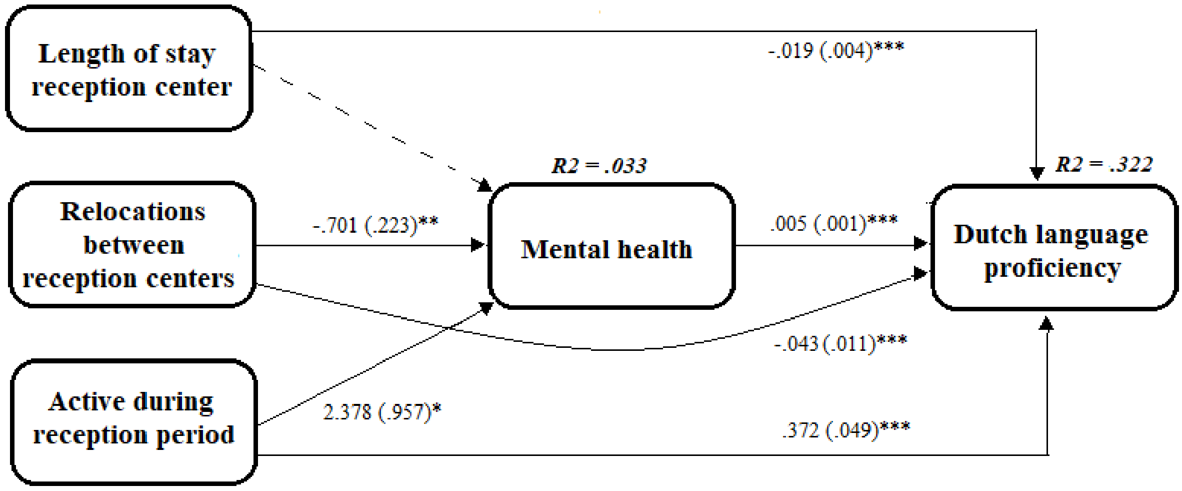

Figure C. A path model explaining the relationship between characteristics of the reception period and host country language proficiency for Syrian permitholders in the Netherlands (*N* = 3023). Unstandardized coefficients with standard errors in the parenthesis. Paths from the control variables gender, age, household characteristics, higher education and length of stay in the Netherlands were accounted for in the model but not reported in the figure and nonsignificant paths are presented as dashed lines. Model fit: (χ^2 (22) = 93.138, *p* = .000, CFI = .980, TLI = .959, RMSEA = .033). * *p* <.05; ** *p* <.01; *** *p* <.001

Online Resource 5 – Alternative analysis including separate dummies for activities during the reception period

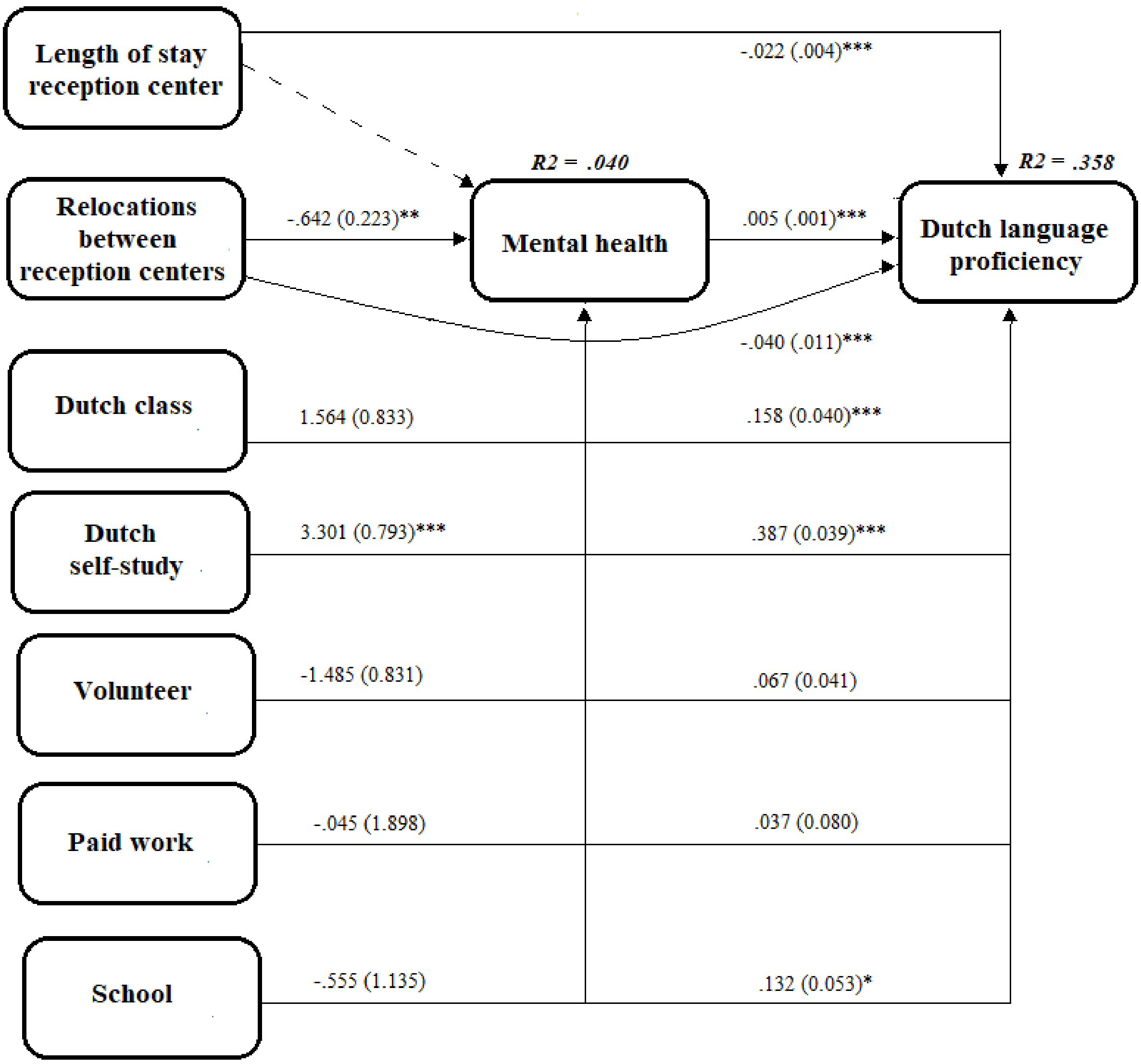

Figure D. A path model explaining the relationship between characteristics of the reception period and host country language proficiency for Syrian permitholders in the Netherlands (*N* = 3023). Unstandardized coefficients with standard errors in the parenthesis. Paths from the control variables gender, age, household characteristics, higher education and length of stay in the Netherlands were accounted for in the model but not reported in the figure and nonsignificant paths are presented as dashed lines. Model fit: (χ^2 (30) = 100.092, p = .000, CFI = .980, TLI = .958, RMSEA = .028). * p <.05; ** p <.01; *** p <.001

Online Resource 6 – Alternative analysis including a cut-off point for length of stay in reception (9 months or more)

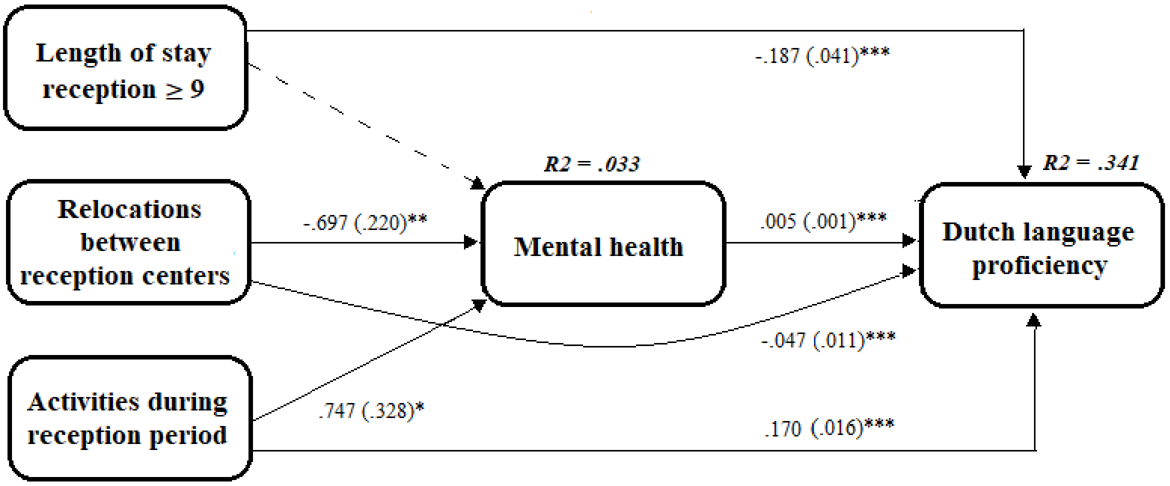

Figure E. A path model explaining the relationship between characteristics of the reception period and host country language proficiency for Syrian permitholders in the Netherlands (N = 3023). Unstandardized coefficients with standard errors in the parenthesis. Paths from the control variables gender, age, household characteristics, higher education and length of stay in the Netherlands were accounted for in the model but not reported in the figure and nonsignificant paths are presented as dashed lines. Model fit: (χ^2 (22) = 97.982, p = .000, CFI = .978, TLI = .955, RMSEA = .034). * p <.05; ** p <.01; *** p <.001

Online Resource 7 – Alternative analysis including a cut-off point for relocations between reception centers (3 relocations or more)

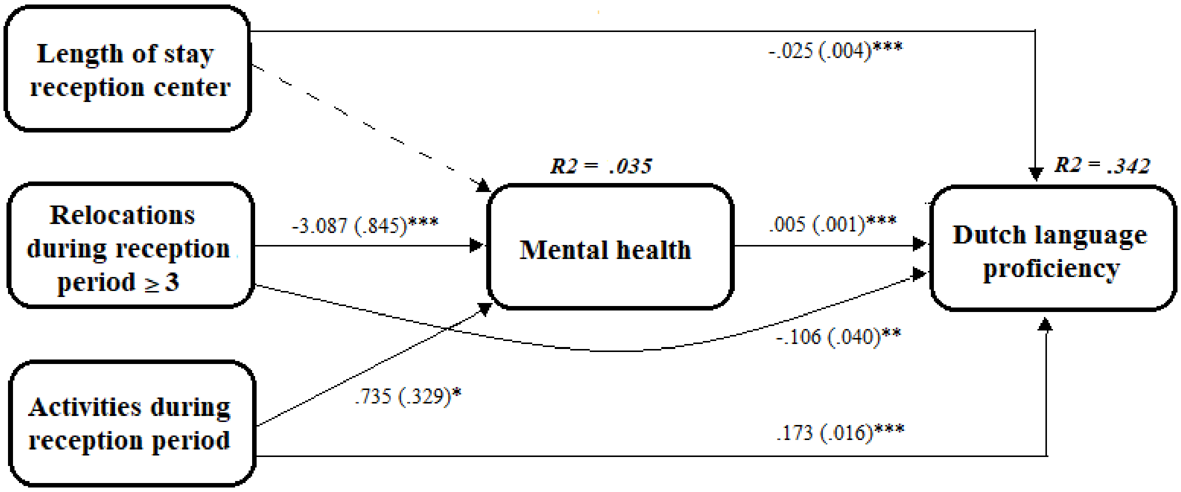

Figure F. A path model explaining the relationship between characteristics of the reception period and host country language proficiency for Syrian permitholders in the Netherlands (*N* = 3023). Unstandardized coefficients with standard errors in the parenthesis. Paths from the control variables gender, age, household characteristics, higher education and length of stay in the Netherlands were accounted for in the model but not reported in the figure and nonsignificant paths are presented as dashed lines. Model fit: (χ^2 (22) = 97.857, *p* = .000, CFI = .978, TLI = .955, RMSEA = .034). * *p* <.05; ** *p* <.01; *** *p* <.001

Online Resource 8 – Alternative analysis including either length of stay in reception center(s) or relocations between reception center(s)

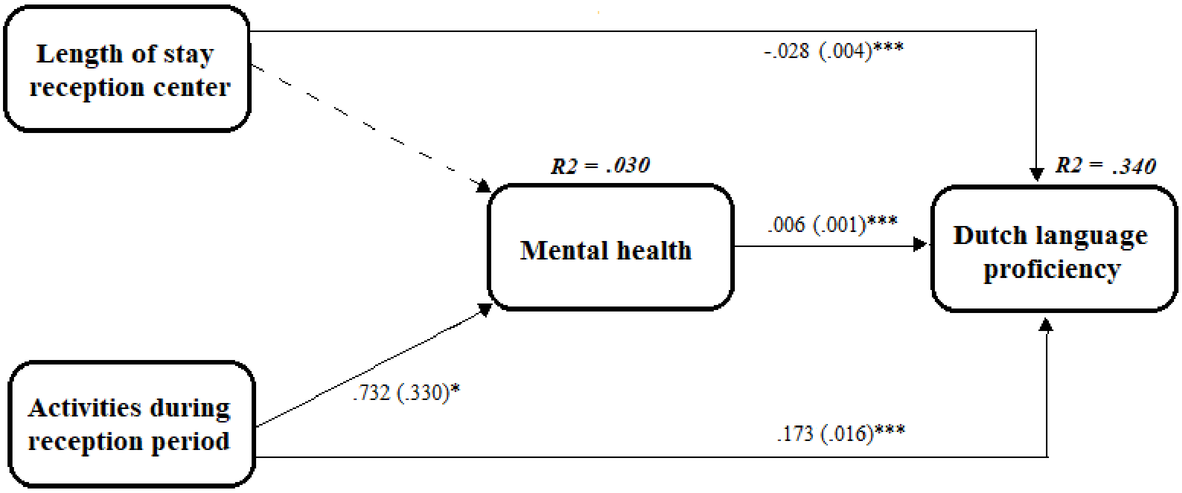

Figure G. A path model explaining the relationship between characteristics of the reception period and host country language proficiency for Syrian permitholders in the Netherlands (N = 3023). Unstandardized coefficients with standard errors in the parenthesis. Paths from the control variables gender, age, household characteristics, higher education and length of stay in the Netherlands were accounted for in the model but not reported in the figure and nonsignificant paths are presented as dashed lines. Model fit: (χ^2 (20) = 97.873, p = .000, CFI = .978, TLI = .954, RMSEA = .036). * p <.05; ** p <.01; *** p <.001

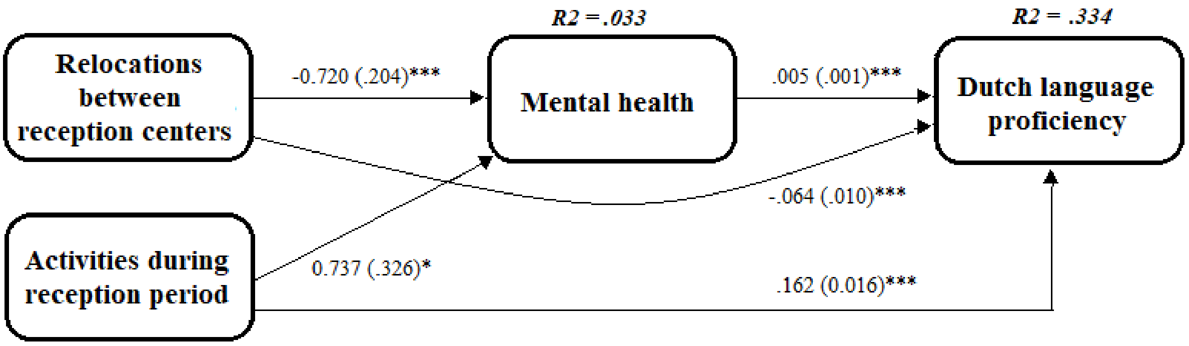

Figure H. A path model explaining the relationship between characteristics of the reception period and host country language proficiency for Syrian permitholders in the Netherlands (N = 3023). Unstandardized coefficients with standard errors in the parenthesis. Paths from the control variables gender, age, household characteristics, higher education and length of stay in the Netherlands were accounted for in the model but not reported in the figure and nonsignificant paths are presented as dashed lines. Model fit: (χ^2 (20) = 93.903, p = .000, CFI = .979, TLI = .957, RMSEA = .035). * p <.05; ** p <.01; *** p <.001

Online Resource 9 – Alternative analysis excluding family members who reunited by proxy

Our sample includes Syrians who received a (temporary) residence permit in the Netherlands between January 1st, 2014 and July 1st, 2016 as well as their children born in the Netherlands and family members who reunited in 2014/2015. Those who reunited often have a different type of reception period as those who came as first applicants, since they for example have the opportunity to move in with their sponsor instead of living in a reception center. For this reason, we checked if our findings held when excluding family members who reunited based on a proxy. As we do not have a variable indicating if one is a family member who reunited or not, we decided to exclude all respondents who had lived in a reception center for one month or shorter (*N=255*) as a proxy for being a family member who reunited.

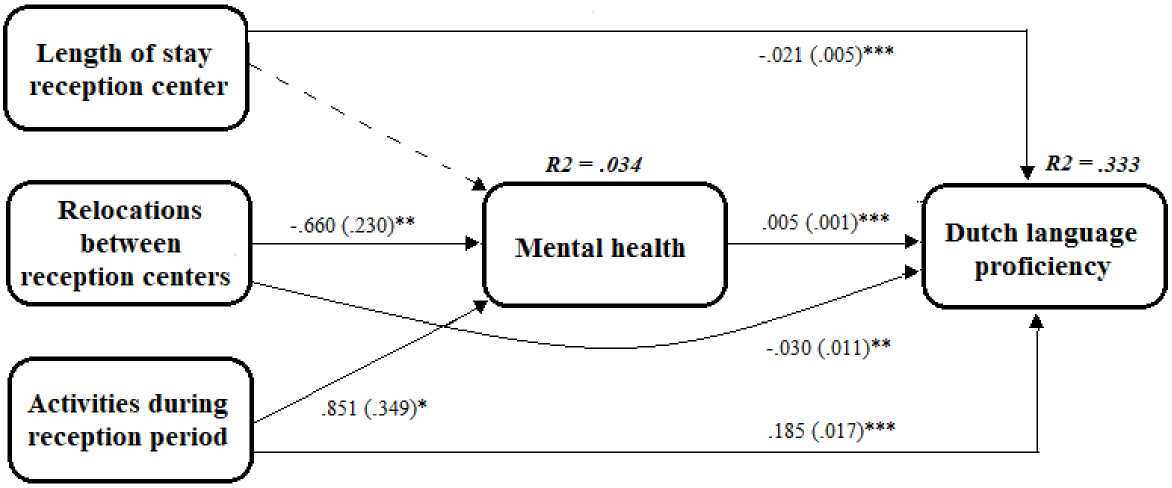

Figure I. A path model explaining the relationship between characteristics of the reception period and host country language proficiency for Syrian permitholders in the Netherlands (*N* = 2768). Unstandardized coefficients with standard errors in the parenthesis. Paths from the control variables gender, age, household characteristics, higher education and length of stay in the Netherlands were accounted for in the model but not reported in the figure and nonsignificant paths are presented as dashed lines. Model fit: (χ^2 (22) = 92.675, *p* = .000, CFI = .978, TLI = .954, RMSEA = .034). * *p* <.05; ** *p* <.01; *** *p* <.001
